# Meta-analysis of studies on chemical, physical and biological agents in the control of *Aedes aegypti*

**DOI:** 10.1186/s12889-015-2199-y

**Published:** 2015-09-04

**Authors:** Estelita Pereira Lima, Marília Oliveira Fonseca Goulart, Modesto Leite Rolim Neto

**Affiliations:** Universidade Federal do Cariri/Faculdade de Medicina do ABC, Av. Príncipe de Gales, 821 - Príncipe de Gales - Santo André / SP - CEP: 09060-650, Rua Divino Salvador, 284, CEP: 63180000 Barbalha, CE Brazil; Universidade Federal de Alagoas, Instituto de Química e Biotecnologia, Universidade Federal de Alagoas, Av. Lourival Melo Mota s/n, Campus Universitário, BR 104, KM 97,6, CEP: 57072900 Maceió, AL Brazil

## Abstract

**Background:**

*Aedes aegypti* is a vector of international concern because it can transmit to humans three important arboviral diseases: yellow fever, dengue and chikungunya. Epidemics that are repeated year after year in a variety of urban centers indicate that there are control failures, allowing the vector to continue expanding.

**Methods:**

To identify the most effective vector control strategies and the factors that contributed to the success or failure of each strategy, we carried out a systematic review with meta-analysis of articles published in 12 databases, from 1974 to the month of December 2013. We evaluated the association between the use of whatever chemical substance, mechanical agent, biological or integrated actions against *A. aegypti* and the control of the vector, as measured by 10 indicators.

**Results:**

We found 2,791 articles, but after careful selection, only 26 studies remained for analysis related to control interventions implemented in 15 countries, with 5 biological, 5 chemical, 3 mechanical and 13 integrated strategies. The comparison among all of them, indicated that the control of *A. aegypti* is significantly associated with the type of strategy used, and that integrated interventions consist of the most effective method for controlling *A. aegypti*.

**Conclusions:**

The most effective control method was the integrated approach, considering the influence of eco-bio-social determinants in the virus-vector-man epidemiological chain, and community involvement, starting with community empowerment as active agents of vector control.

## Background

*Aedes aegypti* is a species of international concern because it can transmit to humans three important arboviral diseases: yellow fever, dengue and chikungunya, which have spread to all continents. The main one of these is still dengue, whose incidence has increased 30 fold in the past 50 years, with increasing geographic expansion to new countries. In the last 10 years it has also expanded to smaller towns and rural areas [1].

In Brazil, *A. aegypti* is found distributed throughout the country, with a circulation record of three serotypes of the dengue virus (DENV) [2], a situation that increases the risk of severe forms of the disease, the lethality rate and the number of deaths [3].

The prevention of dengue depends on the control of its vector to interrupt the chain of transmission and, probably, this strategy will continue to be the main one, even when an effective vaccine against the virus is implemented [4], together with other strategies, such as the blockage of viral transmission by the bacteria *Wolbachia* in infected mosquitoes [5].

In general, vector control can be carried out, using a chemical, physical, biological, or an integrated approach, and the components of this system include entomological surveillance, source reduction (or environmental management), biological control, chemical control, with the use of insecticides and repellents; traps and insecticide resistance management [6].

In Brazil, since 1947, the year diphenyltrichloroethane (DDT) was introduced to Public Health campaigns, synthetic insecticides were adopted as a form of priority vector control [7]. However, the epidemics that are repeated year after year in various urban centers indicate that there are flaws in the control, allowing the vector to continue expanding [8].

Considering all the knowledge produced on combating *A. aegypti*, it is necessary to collect these findings, systematically assessing the gains and losses for each form of control, with the goal of identifying the factors that contribute to the success or failure of the control strategies employed.

## Methods

A Systematic Review Study with Meta-Analysis was carried out, which aimed to combine studies on a given topic without bias, and group the individual data for each one [9].

The searches were done through the Capes Portal of journals (*Portal de periódicos da Capes*) which located articles in the databases, *Scopus, Medline, SciVerse Science Direct, OneFile, Science Citation Index Expanded, Pubmed Central, PLoS, SpringerLink, Directory of Open Access Journals, Dialnet, SciELO,* and *SciELO Brazil.*

Search terms entered were: *Aedes aegypti*, vector control, biological control, larvicidal activity, growth regulator, larvae-eating fish and oviposition traps, written in both languages, Portuguese and English. Peer-reviewed articles, published up to the month of December 2013 in all languages, were selected.

Journal articles on control strategies that did not include field testing, and exclusively on entomological surveillance were excluded.

The evaluated intervention was the use of any chemical substance, physical agent, biological or integrated actions against *A. aegypti*, regardless of the formula, concentration, form of application, target stage of the mosquito and duration of treatment. The outcome analyzed was vector control, measured through the following indicators: infestation indices as represented by household infestation index - HI; Breteau index - BI; pupae per person index - PPI; pupae per house index - PHI, average positivity for house, mosquito density, proportion of eggs collected, mortality index of mosquitoes and rate of viral transmission and incidence of dengue.

Data were statistically analyzed using the program Bio-Stat 5.0, which allowed meta-analysis of the studies using the *p-value* application (*pw*, combined), since the level of significance was the only result comparable and common to 22 of 26 articles analyzed. The *p-value* for each study was converted into a Naperian logarithm, and applying, in the end, a chi-square test for obtaining the combined value (*w = pooled*).

To control publication bias the following steps were adopted: 1. The definition of the formulated question should be clear, direct and objective. 2. The selection of the articles used in the study should be made according to the relevance of the articles and to their relation to the formulated question. 3. The selection of the relevant articles should be in accordance with the inclusion and exclusion criteria. 4. The data compilation should be done by the reading and categorization of the relevant articles. 5. Careful assessment should be made on the relevance of the topic that is being discussed in the selected articles with the formulated question in the study. 6. Careful association should be made among the results of studies, using as a parameter, the relevance of the selected articles with the addressed theme in the research and its scientific evidence. 7. Thematic variation should be made among the studies selected stressing the validity of their evidence in order to answer the formulated question. 8. Assessment must be made of how much of the data from the selected articles may be generalized in order to be used as statistics sources for the methodological design and for the systematic review.

## Results

### Systematic review

**Fig. 1:**
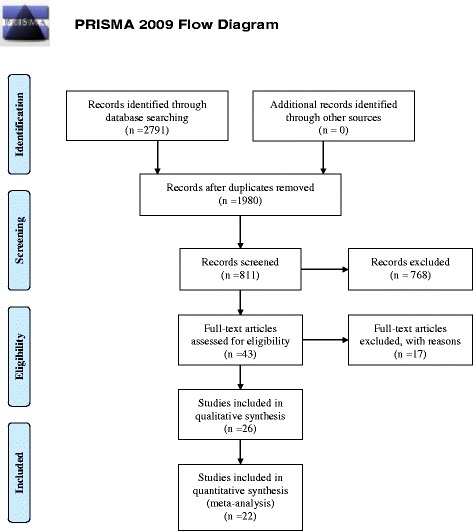
Flow chart showing study selection for the review

A total of 2,791 articles were found, of which 1,980 were duplicates. Analysis of 811 abstracts guided the selection for 43 studies, but after reading the complete articles led to the exclusion of 17 of them, for not following the proposed criteria (Fig. 1). Thus, 26 studies were considered eligible for systematic review and their characteristics are described in Table 1.

**Table 1:** Characteristics and results of 26 reviewed studies

Control Strategy	Country	N	Period of intervention	Main findings	Author, year
Biological					
*Betta splendens*	Brazil	537 containers (1 fish/container) and 437 containers with Bti distributed in a neighborhood.	6 months	The fish remained in 97.6 % of the containers for a period of 45-60 days, but in six months they were only in 13.5 % of these. The vector infestation indices in these containers was 19x lower compared to those with Bti, and their performance 85 % better.	Oliveira-Lima et al. [13]
*Bacillus thuringiensis (Bti)*					
*Poecilia reticulata*	Cambodia	1,626 containers (2–3 fish/container) and 541 containers without fish, distributed in 14 villages.	12 months	The fish remained in 56.9 % of the containers after 12 months. A BI of 71.9 was recorded, while in the containers without fish, the BI was 392.3. The local infestation index was reduced by 79 %.	Seng et al. [15]
*Betta splendens*	Brazil	2,071 tanks (1 fish/tank) distributed in three neighborhoods.	20 months	There was a 320 x decrease in the infestation of the tanks.	Pamplona et al. [14]
*Mesocyclops ssp.*	Vietnam	5,111 containers with *Mesocyclops* and 3,426 with *Micronecta* distributed in three provinces.	120 months	In containers with *Mesocyclops* there was a 50 % reduction in *A. aegypti* infestation and in those with *Micronecta* the protective effect was even greater.	Nam et al. [17]
*Micronecta quadristrigata* Bredd					
*Bacillus thuringiensis* (Bti)	Brazil	140 houses distributed in five neighborhoods.	2 months	Among three commercial brands evaluated, Bactivec® showed better results, because it reduced the HI by 95.4 %, while for G® and WDG® (Vectobac®) brands, the drop in this index varied from 17.5 to 22 %. No reduction was observed in the indices of the control area.	Assumpção Filho and Silva, [19]
Chemical					
Triflumuron	Australia	5 replica buckets (2 L) per treatment (0.48 and 0.96 ppm); 5 replicates with only water.	~6 months	For up to 12 weeks the insecticide inhibited the emergence of adults, and for up to 20 weeks the emergence of pupae remained below 50 %. At the 0.96 ppm dose, the insecticide inhibited the development of pupae, by at least, 50 x the rate of the control during the study period.	Jacups et al. [28]
Permethrin and pyriproxyfen (in ULV and fumigation)	Argentina	5 areas with different treatments:	3 weeks	ULV treatment with 10 % permethrin and 10 % permethrin plus 3 % pyriproxyfen, using the cold fogger truck mount ULV resulted in the greatest number of dead larvae. After the treatments, the lowest value of BI was observed in the area treated with the canister fumigant formula, and a long-lasting effect was observed with a formula of 10 % permethrin and 3 % pyriproxyfen. Three weeks elapsed before the pre-treatment value was restored.	Dantur Juri et al. [26]
		1- 10 % Permethrin in ULV; 2- 10 % permethrin and 3 % pyriproxyfen in a fumigant canister;			
		3- the same formulas applied in area 2, in ULV;			
		4- 10 % permethrin in ULV in portable aerosol pumps;			
		5- without treatment;			
Deltamethrin	Thailand	86 curtains	12 months	The impregnated curtains caused 98.2 % mortality, within 12 months after the intervention. Washing of curtains by hand, the use of detergent and sun exposure did not reduce the residual effect of the insecticide, but the washing machine increased the survival of the mosquito by 6x. The presence of dust in the curtain was a positive factor for the residual effect.	Vanlerberghe et al. [21]
Highcis-Permethrin	Colombia	200 houses were subjected to applications of cis-permethrin and 126 homes to ß-cypermethrin.	6 months	There was a reduction of over 80 % in the density of mosquitoes after 1 day of treatment. After five weeks, the results showed no striking effects.	Castro et al. [25]
ß-cypermethrin					
Fenitrothion (in ULV)	Thailand	1,500 homes in one neighborhood.	18 months Double application at an interval of 14 days	The BI was reduced by 84.8 and 90.7 %, from 8 to 17 months after treatment.	Pant et al. [27]
Physical or mechanical					
Plastic covers for tanks	Sri Lanka	46 tanks with intervention; 46 tanks without intervention.	12 months	The average positive tanks decreased from 10.5/month to 1.17/month after intervention, whereas there was no change in the control.	Kusumawathie et al. [31]
Manual cleaning with bleach and detergent	Honduras	8 neighborhoods underwent intervention and 5 were controls; 1,784 houses were visited.	5 months	The initial evaluation conducted revealed that there was no striking reduction in infestation indices. With an increase in the concentration of the ingredients and the number of pupae and 3rd and 4th instar larvae were significantly lower than in untreated neighborhoods.	Fernández et al. [29]
Ovitraps	United States	330 oviposition traps were installed in 165 homes (2/home); 145 homes remained as the control.	12 months	The BI underwent a decrease of 36 %, while in the control area it increased by 500 %. Reduction in the HI in the intervention area was not significant, but in the control area these indices increased to 440 %.	Cheng et al. [30]
Integrated					
Container covers (Olyset®) impregnated with permethrin	Vietnam	313 houses underwent the intervention and 363 remained as controls; The evaluation of results was done in 3,869 containers in the trial area and 4,198 in the control área.	7 months	After a month of study, the BI was 10.5 in the intervention area, while in the control area it was 41.6.	Tsunoda et al. [10]
Pyriproxyfen					
				5 months after the intervention, the containers for the control showed an infestation 2.3 times higher than in containers with covers. Those treated with pyriproxyfen had a greater reduction in the number of pupae as compared to the controls. Despite that the treatments showed persistence for more than five months, no significant difference was observed in the rate of viral transmission in both areas.	
Lethal ovitraps with Bti and buprofezin	Pakistan	72 ovitraps were installed in 2 municipalities divided among 18 intervention houses, with 36 lethal ovitraps (LO) and 18 controls with 36 ovitraps without any treatment.	4 months	10,152 *A. aegypti* eggs were recovered with 5,351 and 4,801 from treatment and control blocks, respectively, indicating that different treatments did not affect oviposition. Ovitraps with infusion had more eggs (6,548) compared to those with only water (3.604).In ovitraps with Bti (10 and 100 ppm) there was a complete inhibition of pupae, but the buprofezin was more effective in interrupting the pupa-adult cycle. The union of the two larvicides at all concentrations, and in grass infusion was more effective in inhibiting the stages of larva-pupa and pupa-adult.	Jahan and Sawar, [20]
Curtains and container covers treated with deltamethrin	Guatemala	1,835 houses	18 months	6 weeks after intervention (rainy season) the number of pupae increased from 1,173 to 4,477, while in the control area, the increase was from 464 to 4,375 pupae. After the 2^nd^ intervention, the number decreased to 1,032 in the clusters with intervention, and to 3,022 in the controls. The curtains caused 100 % mortality within 18 months, and for the drums they caused 85.3 % mortality in the same period.	Rizzo et al. [22]
Temephos and elimination of breeding grounds		20 clusters; 10 received 3,079 curtains and 298 drum covers;10 remained as the control.	2 interventions (at 2, 17 months after)		
Fish	India	10-15 *Poecilia* fish were released into 514 tanks and the same number of *Gambusia* into 337 tanks; 50 people were surveyed.	4 months	*Poecilia* was the most effective and more resistant fish. After 1 month, the larval density was 0.2 and 7.8 in two areas, with survival rates of 86 and 33.7 %. Only 16 % of *Gambusia* fish survived for the same period, and showed a larval density of 11.7. The use of *Poecilia* integrated with an educational campaign showed significant impact, but the same was not observed when considering the actions separately.	Ghosh et al. [16]
*Poecilia*					
*Gambusia affinis*					
Educational campaign					
Lethal ovitraps with deltamethrin	Pakistan	50 houses received 18 pairs of lethal ovitraps (deltamethrin 2.5, 5 and 20 ppm);	2 and a half months	The treated ovitraps had significantly fewer eggs (189, 87 and 61) compared to untreated controls (1019, 1305 and 949). Ovitraps containing the 20 ppm concentration were most effective for the control of the *Aedes* population.	Jahan et al. [23]
		20 houses received the same amount of ovitraps with distilled water.			
Ovitraps with deltamethrin	Colombia	10 houses with intervention in 4 neighborhoods	24 months with monthly substitution of Bti	No significant differences were observed between treatments, but considering the entomological indices before and after intervention, the HI went from 15.1 to 8.5 %; The average pupae/house went from 1.15 to 0.07 and the adult rate from 56.3 to 34.8 %.	Ocampo et al. [24]
Bti					
Education					
Temephos	Argentina	120,000 houses and 137,000 applications of larvicide every 4 months and applications in ULV, in emergency situations	60 months	The BI decreased significantly in all focus cycles, compared to the pre-intervention period. The incidence of dengue fell from 10.4/10,000 inhabitants in the year 2000 to 0, between the period 2001 and 2006, increasing only to 4.5/10,000 inhabitants in 2007, with the introduction of a different serotype.	Gürtler et al. [11]
Bti					
Pyrethroids					
Elimination of breeding					
Lethal ovitraps (with Bifenthrin) (biodegradable)	Australia	206 lethal ovitraps (4/premise) with insecticide (SLO) were installed. 500 biodegradable ovitraps (4/premise) (BLO); The monitoring was done through 15 sentinel ovitraps (BGSs) and 20 non-lethal ovitraps.	1 month	In the rainy season the number of mosquitoes captured and killed was higher than the dry season, counting 993 females in the SLOs, and 119 in the sentinels, an average number significantly lower than in the control. In 53.2 % of the biodegradable ovitraps a total of 6,654 *A. aegypti* eggs were contained. Over the intervention period, collections of *A. aegypti* in the treatment areas were significantly less than in the control area is BGSs but not SLOs. The two lethal ovitraps were effective in vector control.	Rapley et al. [32]
Bednets treated with permethrin (Olyset®)	Haiti	1,017 houses belonging to 18 clusters; 9 sectors received mosquito nets and 9 were the controls.	12 months	In the 1^st^ month reduction in infestation rates in the intervention area was higher than in the control. After 5 months, the result was the opposite. However, control houses located within 50 m of the mosquito net houses had significantly lower HI and PPI at 1 month, an effect that extended to 100 m by 5 months. After 12 months, the number of dengue cases underwent a reduction of 15.3 %.	Lenhart et al. [12]
Ovitraps with Bti	Brazil	5 areas, from 80 to 100 ovitraps/area were instaled 464 sentinel ovitraps (S-OVT) with Bti (2 g) and 5,602 control ovitraps (C-OVT) with Bti (4 g).	12 months with monthly substitution of paddles, infusion and Bti	4 and 6.3 million eggs through the S-OVTs and C-OVTs were collected, respectively. The use of ovitraps can prevent mosquito population growth, depending on the number of eggs collected, and the increase of Bti also impairs hatching of the eggs.	Regis et al. [33]
Resevoir cleaning	Thailand	966 houses and 5,821 reservoirs inspected.	---	Weekly cleaning was more effective than the monthly and annual cleaning (The proportion of infested containers was 17.2, 39.1 and 43.7 %, respectively); The fish reduced the % of larvae from 43.7 to 7.0 % cement tanks, being the most effective form of all that were evaluated; The covers were also effective, but its effect decreases with the frequency of use of reservoirs; Temephos was effective only in points in the urban area.	Phuanukoonnon et al. [34]
Fish			(Survey)		
Covers					
Temephos					
Malathion	Mexico	187 houses were selected, with 47 submitted to the campaign, 46 to malathion, 49 to 2 interventions, and 45 to no interventions	6 months	The overall average of the positive containers by house was reduced from 0.97 to 0.77. This reduction was more apparent in the houses that were a part of the educational campaign in relation to the ones that received malathion spraying.	Espinoza-Gómez et al. [35]
Educacional Campaign					
*Mesocyclops* and Community participation	Vietnam	37 communes; 309,730 people	72 months (1998–2003)	*A. aegypti* was eliminated from 32 communes. During the years 2000 and 2003, no dengue case was reported in any of the studied areas.	Kay and Nam, [18]

The analyzed articles referred to control interventions implemented in 15 countries and published since 1974. They were classified into biological, chemical, physical or mechanical and integrated approaches. The latter comprises two or more strategies employed simultaneously. Five articles were considered eligible for the biological group, five for the chemical group, three for physical or mechanical and 13 for the integrated group. The time interval for the interventions ranged from 2 weeks to 72 months.

The action of six biological control agents were compared: three species of fish, crustaceans, aquatic insects and the bacteria-based larvicide *Bacillus thuringiensis var israelensis* (Bti); nine chemical insecticides belonging to the classes: pyrethroids, organophosphates, benzoylureas, phenyl ether and thioridazine; three physical or mechanical agents: regular cleaning of containers, container covers and ovitraps. Some of these agents were applied together in integrated vector control programs.

The most widely used biological control agents were fish and Bti. The species of fish involved were *Betta splendens*, *Gambusia affinis* and *Poecilia reticulata*. Different brands of Bti were evaluated in containers for domestic use and in ovitraps.

Control performance was evaluated based on the infestation indices (HI, BI, PPI, PHI), average positivity for home, mosquito density, proportion of eggs collected, mosquito mortality rate, rate of virus transmission and incidence of dengue. Of the 26 articles analyzed, only three mentioned indicators of impact on dengue: one from Vietnam, one in Argentina and another one in Haiti [10–12].

The performance of *Betta splendens* was individually evaluated and compared to Bti (Vectobac®), in Brazil. One field test used fish in 537 containers, distributed in a neighborhood in the city of Fortaleza - CE, with continuous evaluation over 6 months. In parallel, 437 containers were treated with Bti. After 60 days of evaluation, 97.6 % of the fish were still present in the containers, leading to a vector infestation 19 times lower than in those with Bti [13]. Another study, also conducted in Brazil, using 2,071 tanks (1 fish/tank) had shown that *Betta* fish were able to reduce the infestation indices in tanks by 320 times [14].

The fish *Poecilia reticulata*, commonly known as a guppy, was introduced into 1,626 containers (2–3 fish/container) distributed in 14 villages in Cambodia. Fish reposition could be made, as necessary. One year after project commencement, about 57 % of the containers still had fish present, indicating a 79 % reduction in vector infestation in the study area, with a recorded BI of 71.9 while in untreated containers that index was 392.3 [15].

In another study, the performance of *Poecilia reticulata* against *Gambusia affinis* was compared in the Tumkur and Kolar districts of India. The two fish species were introduced into cement tanks (10 to 15 fish/tank). *Poecilia reticulata* species were released into 482 tanks in the village of Domatmari and 32 tanks in Srinivaspura village (Tumkur), whereas *Gambusia* was introduced into 337 tanks in Balmanda village (Kolar). *Poecilia* showed superior resistance and a fall in larval density. One month after the fish were introduced, 86 % were still present in tanks in Domatmari and 33.7 % in Srinivaspura, while in Balmanda only 16 % of the tanks contained fish, however, the significant impact on the control of *A. aegypti* was associated with concomitant educational campaigns. The same was not observed when considering the control measures separately [16].

In three provinces in northern Vietnam, the action of copepods, *Mesocyclops ssp*. and *Micronecta quadristrigata* Bredd, and small aquatic insects were evaluated. These larvae predators were found in natural and artificial habitats, within the communities. After laboratory assays, the predators were distributed to the community using 5,111 containers with *Mesocyclops* and 3,426 with *Micronecta* notification and then evaluated. The copepods promoted a 50 % reduction in infestation of *A. aegypti* in the containers, while the use of *Micronecta* promoted a greater protective effect [17].

Due to the success of this biological control strategy with copepods in Vietnam, it was replicated out elsewhere in the country, reaching *A. aegypti* elimination in 32 communities, with no dengue reports in those areas for 1 year [18].

Evaluations on the residual effects for different commercial formulas of Bti were carried out in Brazil, in 140 houses, distributed in five neighborhoods of Nova Iguaçu-RJ. Bactivec® showed the highest residual effect (62 days), contributing to a reduction in HI of 95.4 %, while (Vectobac®) reached only a 22 % reduction [19].

In another study conducted in Brazil, Bti was added to ovitraps (2:04 g/trap) with hay infusion, and installed in five urban landscapes of the city of Recife-PE. The objective was to evaluate the use of ovitraps as a surveillance tool and for control of *A. aegypti*. There were 464 sentinel ovitraps installed (80–100 area) treated with 2 g of Bti, and 5,602 control ovitraps with 4 g of larvicide. In the ovitraps with Bti (2 g) a monthly change of paddles, Bti and hay infusion was carried out, but in those with higher concentrations, Bti was replaced every 2 months. During 12 months of follow-up 10.3 million eggs were collected from the two ovitraps, with no hatching of larvae observed, indicating that the addition of Bti to ovitraps increases the action of the traps.

In two municipalities of Pakistan, Bti efficiency was evaluated for ovitraps alone or associated with the growth regulator buprofezin. Seventy-two ovitraps were distributed in 18 houses within the municipalities. Thirty-six of them were treated with two concentrations of Bti (10 and 100 ppm). Both completely inhibited the formation of pupae in the ovitraps. Their effectiveness was greater in the inhibition of larval stages to adult when combined with the growth regulator buprofezin and grass infusion. In comparing Bti to buprofezin, the latter was more effective in interrupting the pupa-adult cycle [20].

Regarding chemical controls, field tests using insecticides, alone or in combination with other control strategies were evaluated. Mechanical barriers like ovitraps, covers, curtains, and mosquitoes nets were used. Of the articles that addressed the individual action of insecticides, six referred to pyrethroids; three to organophosphates, one to benzoylurea, and one to thioridazine. Three articles assessed the joint action of two or more insecticides.

The pyrethroid deltamethrin showed the highest residual effect and was more effective when impregnated in curtains (PermaNet®). In Guatemala, the curtains caused 100 % mortality in adults within 18 months after the intervention, and 98.2 % mortality in Thailand, after 12 months [21, 22]. A similar result was observed for container covers (85.3 % mortality in 18 months) [22].

Impregnation of bednets (Olyset®) with the pyrethroid permethrin in Haiti [12], did not show an effect as lasting as curtains and covers impregnated with deltamethrin [21, 22]. The strategy applied in Haiti, in 1,017 houses, led to the reduction of infestation indices in the area of intervention, when compared to the control area, for up to 5 months. However, after this period, a reversal of results was obtained. HI, CI and BI were lower in the control trap than in the bednet trap [12].

In Pakistan, a study conducted in 50 houses, was carried out to compare the effect of three concentrations of deltamethrin (2.5, 5 and 20 ppm) with other untreated ovitraps. For the treated ovitraps, the number of eggs collected was significantly lower (189, 87 and 61) compared to the control (1019, 1305 and 949) [23]. However, when compared to ovitraps treated with Bti, in Colombia, no significant difference at any level was observed among the treatments [24].

The pyrethroid permethrin, when impregnated in container covers (Olyset®) also showed satisfactory results in a study conducted in 676 houses in Vietnam. Five months after the intervention, the control containers had an infestation 2.3 times greater than in those with covers [10]. However, in applications of ultra-low volume (ULV) including fumigation, its effect was not long lasting. In Putumayo-Colombia, high levels of cis-permethrin and ß-cypermethrin were applied to ovitraps and placed in 200 and 126 houses, respectively, following the recommended protocol in the case of a dengue outbreak (3 applications, one each for 3 days). The mortality in the sentinel traps exposed to high *cis*-permethrin was greater than 75 %, while the others, exposed only to ß-cypermethrin, was around 88 % after one day of treatment. In the same period, this treatment promoted a reduction of over 80 % in the density of mosquitoes, however this effect was not persistent [25].

A similar approach was also applied in Argentina, where permethrin (10 %) alone was evaluated, and/or in combination with pyriproxyfen (3 %). This study also evaluated the form of dispersion: ULV vs. fumigation. The best results in terms of larvae mortality were obtained using the combined treatment at ultra-low volume, for up to 2 weeks after the treatment. However, the effect was not long lasting and the entomological pretreatment indices restored themselves over time [26].

Among the organophosphates, whose individual effects were evaluated, fenitrothion performed better. Two applications of the product and placed in 1,500 homes in one neighborhood of Thailand, resulted in a BI reduction of 84.8 and 90.7 % in 8 and 17 months after treatment, respectively [27].

Among the benzoylurea group, a field test conducted in Australia, using buckets (2 L) (with what inside) revealed that triflumuron inhibited the emergence of adults for up to 12 weeks, and the emergence of pupae remained below 50 % for up to 20 weeks. At the highest dose tested (0.96 ppm), the growth regulator inhibited the development of pupae at least 50 times the rate of the controls over 22 weeks [28].

Only three articles addressed control methods that were exclusively mechanical or physical. The first consisted of regular cleaning of containers with a home-made paste made with detergent and bleach. This study was conducted in Honduras, in 1,784 houses, distributed amongst 8 neighborhoods. The first evaluation demonstrated that the paste at the indicated concentration (5 tablespoons of bleach and 1 tablespoon of detergent) did not have the expected effect. After reformulating the paste (1:2 bags of chlorine bleach plus 1:2 bags detergent instead of 5 tablespoons of chlorine bleach plus 1 tablespoon of detergent), and re-orienting the community, they were able to significantly reduce the number of larvae and pupae compared to the control [29].

The other two methods consisted of collecting eggs in oviposition traps and in covers of containers. Both reduced the entomological indices in the intervention areas, while in control areas there was an increase. In 165 homes in the United States 330 ovitraps were established and evaluated over a period of 12 months. In areas without ovitraps the HI and BI increased 440 and 500 %, respectively [30]. For the container covers, on 46 tanks, in houses in Sri Lanka, the average monthly positivity changed from 10.5/month to 1.17/month, after intervention [31].

Regarding the association of different control measures, these are characterized by joint actions involving one of the following strategies: education, environmental management, use of mixed strategies: biological and chemical compounds belonging to one or more classes; environmental management and mechanical barriers such as container covers, curtains, mosquito nets and ovitraps impregnated with insecticide or not. These interventions were applied in Vietnam [10, 18], Pakistan [23], Guatemala [22], India [16], Colombia [24], Argentina [11], Australia [32], Haiti [12], Brazil [33], Thailand [34] and Mexico [35].

Of the 13 articles that gathered integrated actions, nine of them had a physical barrier against mosquitoes. For six of the studies, community participation (education, elimination of mosquitoes, breeding, etc) was associated with other efforts [11, 16, 18, 22, 24, 36] simultaneously. In five interventions, chemical or biological insecticides were added to ovitraps [20, 23, 24, 32, 33], and in three, the agents were impregnated in curtains, bednets or covers [10, 12, 22].

Considering performance and persistence, integrated intervention with the greatest positive impact was in Argentina. Combat against mosquitoes in the immature stage was done by the chemical larvicide temephos, by biological Bti, with applications every 4 months and elimination of breeding potentials. To control adults, pyrethroid insecticides at ULV were employed in emergency situations. In this study, 120,000 houses were evaluated, with 137,000 applications of larvicides. Over 60 months (2001–2006), BI decreased significantly in all focus cycles, compared to the pre-intervention period, and the incidence of dengue dropped from 10.4/10,000 inhabitants to 0. New cases only appeared again in 2007, upon introduction of a new viral sorotype [11].

### Meta-analysis

Of the 26 eligible studies, four did not have enough information to perform the meta-analysis, or did not have enough data to compare subgroups, leaving, therefore, just 22 articles.

Table 2 shows the combined results of agents used within their own category and overall. The global analysis indicates that all categories of intervention employed contributed significantly to the control of *A. aegypti* (chi-squared (w) = 277.3397 and p (w) <0.0001), but among all the strategies analyzed, it was the integrated intervention that showed the greatest impact (chi-squared (w) = 140.0351 and p (w) <0.0001).

**Table 2:** Performance analysis of control strategies, both isolated and combined, based on the levels of significance of each study (*p*-value)

	Strategies			
Statistics	Biological	Chemical	Integrated	Global
Number of studies	5^a^	5^b^	12	22
Chi-square (w)	72.5071	57.2704	140.0351	277.3397
Degrees of freedom	10	10	24	44
*p*-value (w)	<0.0001	< 0.0001	< 0.0001	< 0.0001

^a^For the small amount of articles (<5) for the biological subgroup meta-analysis, an article about fish x educational campaign was considered as biological

^b^One of the studies tested permethrin Cis and Beta, providing two *p*-values. The two values were used for the meta-analysis of subgroups (covering 4–5 articles)

## Discussion

The effectiveness of interventions for control of *A. aegypti*, implemented in 15 countries, was analyzed through a systematic review with a meta-analysis, in order to evaluate the success of these strategies, and identify which of these performed better.

The control of *A. aegypti* is a complex task, because the permanence of this vector on the human environment is associated with local eco-bio-social factors. Among these factors, the lack of infrastructure in urban centers and the difficulties in securing water supplies and regular garbage collection represented a major challenge. When not overcome, this confines the city to a permanent state of vulnerability, conditioning the population to offer mosquitoes highly productive breeding grounds, such as drums and water storage tanks, coupled with large quantities of artificial breeding grounds, such as plastic bottles, cans and other receptacles [36].

Of all the strategies analyzed those applied in an integrated form represented the most effective control. The World Health Organization (WHO) recommends Integrated Vector Management (IVM) as an ideal control program. It is defined as “a rational decision-making process for the optimal use of resources for vector control. The approach seeks to improve the efficacy, cost-effectiveness, ecological soundness and sustainability of disease-vector control” [37].

In the studies analyzed, only one described a control strategy similar to the IVM program as proposed by the WHO, incorporating four elements: (1) a combined vertical and horizontal approach that depends on community understanding; (2) prioritized control, according to the larval productivity of major habitat types; (3) use of a predacious biological control agent; delivered by (4) community activities of health volunteers, schools, and the public [18].

Regards other integrated approaches, even without fulfilling all the criteria for IVM, the concomitant adoption of different means of eliminating *A. aegypti* assured the best performance. This supports the eco-bio-social context in which the vector is inserted, i.e. the adoption of a single control agent may not have had the expected effect. It is unlikely that a chemical, physical or biological agent is suitable for all types of mosquito breeding sites, or is adapted to whatever the environmental condition is.

A multicenter study done in Asia was conducted between 2006 and 2011 in urban and peri-urban areas of six countries, and where it was considered an eco-bio-social approach, generated evidence relevant to the adoption of controls based on the principles of IVM. The results suggest that for a more sustainable control there should be involvement of several partners, including the local community; the interventions should be directed at a significant reduction of infestation in breeding areas; adoption of new non-insecticidal tools such as lids or container covers and predators of the vector [4].

Corroborating the above authors, a meta-analysis of interventions for vector control of dengue in developing countries, the current authors found that the integrated management was the most effective in reducing HI, BI and infestations in containers [38]. However, in contrast, another review [39] pointed out biological interventions as the most successful and sustainable, according to Mulla’s percent reductions (100–(Control 1⁄Treated · Treated 2 ⁄Control 2) · 100).

In this review, successful strategies by biological control agents was also observed, but it is difficult to pinpoint the most effective agent because the studies lacked standardized predictor variables, for example, species, number of specimens and frequency of replacement of containers, physical-chemical quality of the water and time monitoring. However, one can see that among the fish, *Betta* showed a superior performance to *Poecilia* [13–15], mainly against resistance. Studies performed in Brazil, employing fish/containers, and reposition of fish was programmed according to the visits of health agents, once every 2 months, in the majority of cases. After 6 months the presence of fish in the containers had been reduced to 13.5 % [13, 14], due, probably, to the long interval between visits and inefficient reposition.

In the studies with *Poecilia*, the amount of fish/container was greater (2–3/container, in Cambodia and 10–15/container in India) and reposition could be performed when necessary. Thus, after one year, the fish were still present in 56.9 and 86 % of the containers, respectively [15, 16].

For the adoption of biological control interventions, local cultural, social and environmental characteristics should be considered, because the success of the strategy is affected by such factors as: population adherence, types of water storage containers, water quality and species adaptation to climatic conditions.

The reviews of Ballenger-Browning and Elder [39] pointed out the advantages and limitations of biological control. It was found that bacteria and copepods were well tolerated by the community, lethal only to the target vector, and not contaminating the environment as insecticides do. To corroborate thisstatement, there is as an example of a successful strategy employed in Vietnam, where endemic species of copepods and fish were selected, from natural and artificial national reservoirs [17].

The main disadvantages are associated with the intense work to maintain the organisms in the containers that depend on the above environmental factors, in addition to the emptying of reservoirs, escape or death of the organisms [39]. It should be emphasized that communities are usually against using fish in their drinking containers.

In the case of fish, one of the most influential environmental problems on performance of a species is its resistance to temperature and to the physicochemical characteristics of the water, especially to chlorine. Studies in Brazil evaluating the resistance of *Betta* to these factors revealed that, under laboratory conditions, the fish survived without any discomfort at a chlorine concentration of 1.0 mg/L and 75 % of them survived at a concentration of 1.50 mg/L [40]. Under field conditions, over 97 % of them survived for a period of 45 to 60 days [13].

Regarding tolerance to the larvicides used in the routine program, *Betta* showed greater resistance compared to the larvae-eating fish species *Trichogaster trichopterus* and *Poecilia reticulata* [41].

Studies comparing the action of Bti, a larvicidal produced from the bacteria *Bacillus thuringiensis* with *Betta* and chemical larvicides showed a less effective action of this product [13, 20]. We also realized that the commercial brand of Bti had a strong influence on the larvicidal effect, with Bactivec® being more effective than Vectobac® [19]. Bti showed good results in association with ovitraps, with incremental action, making the collecting network safer for the human being and more lethal to the mosquitoes [20, 33]. Just like any other form of biological control, the use of Bti in ovitraps also requires more frequent maintenance, due to its low persistence.

The longevity of an intervention is of utmost importance to public health, especially those that prioritize the use of insecticides, because those that were short-lived generate a series of negative economic, social and operational effects, such as increased human exposure to these products, increasedwaste disposal impacts on the environment and selection for more resistant mosquitoes.

Evaluations of programs that used only insecticides indicated that few succeeded for more than a year, except in Thailand in the ‘70. The best performance was achieved using the organophosphate fenitrothion at ULV, which lowered the BI to 90.7 % after 17 months [27]. As this is an intervention made for over 40 years, many factors may have influenced this result, e.g. the absence of resistance of *Aedes* to organophosphates, which is currently present in various parts of the world.

In the latest programs, triflumuron, a growth regulator belonging to the benzoylurea group showed the best performance, inhibiting the emergence of adult mosquitoes for up to 12 weeks. However, the field test was conducted with a small sample (5 replica buckets of 2 L each) [28]. Permethrin and pyriproxyfen (ULV and fumigation) were effective, except that after three weeks, vector infestation indexes, observed before the intervention, were restored [26].

Special applications (spraying) at ULV continued to be indicated for emergency situations [38], but several studies showed that the success of these interventions is associated with community involvement [39].

Studies show that the use of insecticides for some years is being criticized, especially regarding spatial application, partially explained by its negative impacts on environmental and human health, in addition to widespread insecticide resistance in the vector. This resulted in a change in policy for the use of insecticides, which led to the prioritization of focal treatment and impregnation of materials used as physical barriers (curtains, container covers, bednets, etc.). These protection measures generate an additional benefit, because they can also protect residents against the vectors of other diseases, such as malaria, leishmaniasis and Chagas’s disease [38, 42].

In these cases, it is observed that the use of insecticides impregnated in some materials obtained more success than when used alone. On curtains for example, deltamethrin caused mortality of adults for as long as 18 months [22] and in container covers treated with permethrin, the treatment persisted for more than 5 months [10].

In this review, we noticed that in several studies, even in the absence of analysis of the effect of community involvement, the implementation of some measures was shown to be directly dependent on community participation, e.g. elimination of breeding places, maintenance and care of fish [13–16, 29], curtains [21, 22], lids and covers [10, 32, 34].

In three studies, the effectiveness of community involvement was assessed in the form of an educational campaign, associated with or compared to other interventions [16, 24, 35]. The measure evaluated alone did not show significant results, but when associated, it contributed to the reduction of vector infestation or enhanced the effectiveness of other interventions. This corroborates the findings of other authors when they suggest that social mobilization is essential for the sustainability of control methods [39].

Some countries have experienced the empowerment of the community in vector control and obtained satisfactory results, with the programs of greatest impact, being those implemented in Vietnam, which reported the expansion of a community-based strategy from 6 to 46 communities, resulting in the elimination of the vector in 40 of the communities, and in Cuba, where for 2 years the BI remained 53 % lower as compared to areas without intervention [43, 44].

Of the integrated control strategies, two are noticeable. The first one implemented in Argentina, based on the adoption of three different classes of insecticides, two being larvicides, one biological and one chemical (Bti and temephos, respectively), complemented with environmental management and the application of adulticides in ULV (pyrethroids) showed a result that was the most impactful in reducing the incidence of dengue for a more extended period of time (60 months) [11]. The second one, implemented in Vietnam, stood out because it promoted vector control in a sustainable way and for an extended time, using the available natural resources, i.e. endemic mosquitoes larvae predators in aquatic collections and community participation, either civil, scholar or health agents. Because the copepods were known by the population, the adhering to the program facilitated both their implementation and maintenance [18].

Assessing the effectiveness of a control program has to consider the indicators of success of the strategy within the eco-bio-social context. Otherwise, the simple reproducibility of a program that was successful in one location may not provide the same results in another. This may explain the discrepancy observed in some articles on the use of similar control measures, but with different results.

Considering the results of this meta-analysis as parameters to evaluate the performance of biological, chemical and integrated control strategies, we observed that the chemical controls alone showed the worst performance, while the integrated strategy showed the best.

### Limitations

Our difficulties are similar to other review studies [38, 39]. The major one being the variability of analysis variables, both as predictors and as outcomes, making it difficult to compare the effectiveness of control measures evaluated. Few studies were randomized and not all had a control.

Regards measures of association, some used odds ratio and confidence intervals; others reported means with or without standard deviations; others, only reported the values of the statistical tests performed with significance levels, and we even found some without any of the aforementioned measures. Because these were treated as important experiences, whose discovered failures also contributed to knowledge in the area of vector control, and published in journals of international recognition, we decided not to exclude them from the systematic review. However, most did not meet the criteria required by traditional meta-analysis methods like random effect (DerSimonia-Laird), or fixed for continuous data or Mantel-Ranszel and Odds ratio. For this reason we opted for the method of combining significance levels (combined pw) and submitted to the process, those that contained such measures.

Another difficulty involved in making comparisons was the sample size and the intervention period. It was noticed that this variation is influenced by the type of intervention tested and by the study model conducted. Therefore, we preferred to indicate as much information as possible regarding the sample, because while most studies referred to a sample unit as one container, a house as one treated material, in another, this unit was an entire area, without reference to the total number of participants of that area.

It is also worth pointing out the variability between entomological indices available in the articles. Many of the researchers adopted BI, HI and the proportion of positive containers as key measures of analysis, but besides these, others were adopted too: pupae per person index - PPI; Index of pupae per house - HPI; average positivity per home, mosquito density, proportion of eggs collected, mortality rate of mosquitoes, viral transmission rate and incidence of dengue. The latter two rates were rarely mentioned in the studies.

It is known that, in most cases the studies aimed at testing interventions such as vector control, but the greater meaning of this is the impact that can be generated for control of diseases. Thus, if studies contextualize their entomological results with rates related to diseases, their analyses would be greatly enriched.

So that future review studies and meta-analysis can be conducted with more precision, we suggest that researchers choose analysis measures and forms of presentations commonly adopted by the scientific community, e.g. Odds ratio with confidence intervals and significance levels (*p*-value); average indices and standard deviations. It is known that each study has its own characteristics, but a greater effort to adopt the most representative indices is essential. Currently, there is a trend in the replacement of infestation indices based on larval counting, for indices that consider the number of pupae, due to the high correlation between pupae and adult mosquitoes, in addition to facilitating species identification and counting of individuals [39, 45].

Finally, we recognize that the minimum amount of studies on mechanical or physical control, may have occurred as a consequence of not including an appropriate descriptor, such as for example, environmental management. In addition, we realize the need that new forms of control should be included in a forthcoming review, based on morphological and genetic manipulation of mosquitoes, as well as ecological interactions between species.

## Conclusions

In conclusion, despite the adversities encountered in the analysis, we believe that integrated interventions consist of the most effective method for the control of *A. aegypti.* The success of this strategy occurs because it enables multidisciplinary and multisectoral involvement, working not only in the direct elimination of the vector, but in the correction of social and environmental shortcomings that contribute to their proliferation. We found that community participation improved all interventions employed associated with it and so it is an indispensable component in any control program.

The most successful integrated strategies used different approaches to attack *A. aegypti.* Considering the main ones, one used insecticides, another, natural predators, but both had components in common: the community involvement, not only as receivers of information, but as active agents of vector control, and environmental management, thereby recognizing the influence of eco-bio-social determinants in the virus-vector-human epidemiological chain. These components, probably ensured the sustainability of programs, and on the basis of the results achieved, we suggest the adoption of integrated control of *A. aegypti* including these factors.

## Abbreviations

HI: Household infestation index (percentage of houses infested with larvae and/or pupae)
BI: Breteau index (number of positive containers per 100 houses inspected)
PPI: Pupae per person index – PPI (number of pupae collected per number of inhabitants of the households inspected)
PHI: Pupae per house index (number of pupae per 100 houses inspected)
